# Dengue pancreatitis with ketoacidosis—a rare manifestation in Endemicity

**DOI:** 10.1093/omcr/omae148

**Published:** 2024-12-10

**Authors:** Kalyan Kumar Das, Rajdeep Basu

**Affiliations:** Department of General Medicine, Siliguri District Hospital, Ward 17, Hakim Para, Siliguri, Darjeeling, West Bengal 734001, India; Department of Endocrinology, Nil Ratan Sircar Medical College and Hospital, 138, Acharya Jagdish Chandra Bose Road, Sealdah, Raja Bazar, Kolkata, West Bengal 700014, India

**Keywords:** Flaviviridae, dengue fever, acute pancreatitis, euglycemic pancreatic ketoacidosis

## Abstract

Dengue may begin as a straightforward fever episode with or without rash also it may affect some organs and cause serious symptoms. Although it is uncommon, severe abdominal discomfort has been a concerning indicator of acute pancreatitis. We report on a male patient, age 26, who first presented with fever, vomiting, and stomach pain. Later, he developed severe pancreatitis. When an acute viral infection causes pancreatitis, ketoacidosis can occur without hyperglycemia. We emphasize that in dengue patients reporting stomach pain, it is important to rule out acute pancreatitis (AP), an uncommon but serious impediment in the course of treatment.

## Introduction

As we know in tropical areas mosquito-borne infections are quite common and dengue fever (DF) is one of them caused by dengue virus (*Flaviviridae)* through the bites of *Aedes aegypti* and *albopictus*. Dengue hemorrhagic fever (DHF) and dengue shock syndrome (DSS) can be manifested as plasma leakage followed by fluid collection in a third space with dyspnea, hemorrhage, or organ-related catastrophe [1]. Some well-known severe manifestations of dengue are myocarditis, Guillain-Barré syndrome, encephalitis, hepatic failure, acute kidney injury, and hemophagocytic syndrome [2, 3]. Another unconventional side effect of dengue fever is acute pancreatitis, primarily linked to severe dengue hemorrhagic fever, Acute pancreatitis can rarely develop ketoacidosis in acute viral infection due to peripheral lipolysis caused by elevated lipase levels in the absence of diabetes mellitus [4].

## Case description

After 3 days of fever a 26-year-old male patient presented to our department of medicine. That sudden onset, continuous, high-grade fever was associated with retroorbital pain, headache, and severe malaise without having a rash. The patient denied any other co-morbidity. After 2 days he developed epigastric pain which was gradually progressive and radiating towards his back and caused nausea and vomiting.

On physical examination, the patient had tenderness in the epigastrium and left hypochondrium. His bowel sounds were sluggish; sensorium and vitals were within normal limits and was febrile. He had an enlarged palpable liver on abdominal examination. Based on his clinical findings, we ordered amylase, lipase, liver function test, renal function test, lipid profile, electrolytes, and ultrasonography whole abdomen. During the workup of fever on day 1, he was found to have dengue NS1 reactive (ELISA-based antigen detection) and other causes of febrile illnesses including malaria (parasite lactate dehydrogenase antigen test and peripheral smear), typhoid (typhi dot IgM), viral hepatitis (HBsAg, Anti HCV) was non-contributory. On day 4, his biochemical parameters revealed transaminitis SGOT (serum glutamic-oxaloacetic transaminase) 622 U/l, SGPT (Serum Glutamic Pyruvic Transaminase) 396 U/l, and hyperlipidemia 489 U/l, and RFT was normal and diagnosed as acute pancreatitis. His laboratory parameters are given below in Table 1 during his hospital stay.

**Table 1:** Complete blood count and other biochemical parameters.

**Parameters**	**Day 1**	**Day 2**	**Day 3**	**Day 4**	**Day 5**	**Day 6**	**Day 7**	**Day 8**	**Day 9**
Hb (gm%)	13.4	13.2	12.6	12.6	13.5	13.4	12.7	13.1	13.4
Hct	34.9	42.1	38.5	38.3	43.3	41.2	39.6	41.0	39.8
TLC (/μl)	4400	3000	3500	3700	4700	6100	6500	6800	6800
Platelet (×10^3^/μl)	185	**130**	**99**	**60**	**80**	150	152	169	343
T. Bil (mg/dl)					1.2	0.8	1.6	1.2	1.1
SGOT (U/l)					**622**	**490**	**293**	**201**	**122**
SGPT (U/l)					**396**	**295**	**206**	**167**	**87**
FBS (mg/dl)	**105**				**104**				**106**
Amylase (U/l)					**90**				**87**
Lipase (U/l)					**489**		**260**	**187**	**99**
Urea (mg/dl)			47		24	22		24	23
Creatinine (mg/dl)			1.1		0.8	0.9		0.9	0.8
TG (mg/dl)						460	400		412
LDL						37	76		57
Na^+^			138			140	136		
K^+^			4.2			3.6	5.3		

**Figure 1:**
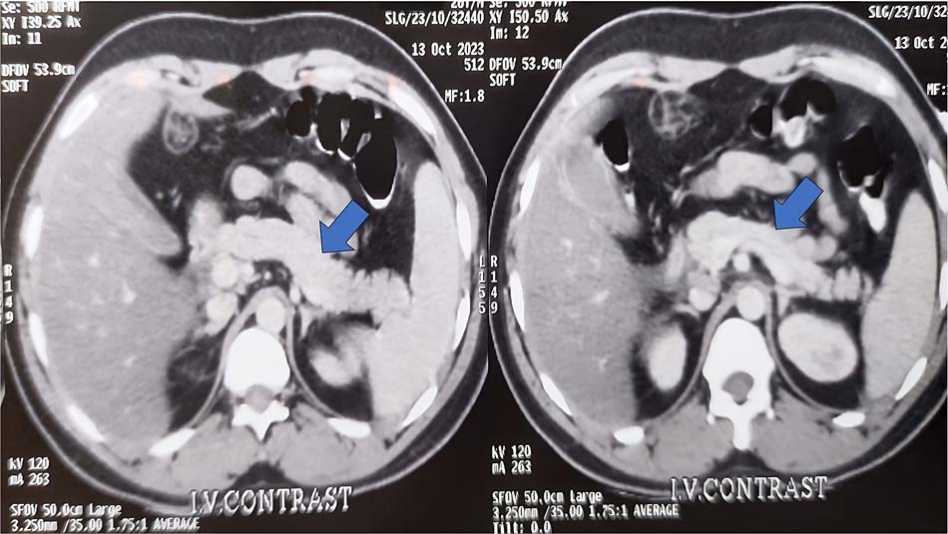
Contrast-enhanced computed tomography of the abdomen shows hepatosplenomegaly and a bulky pancreas with peripancreatic fat stranding and collection with thickening at the right anterior conal fascia and lateral conal fascia.

Further investigations reveal a mildly enlarged liver with a bulky pancreas on ultrasonography. His contrast-enhanced CT scan abdomen reveals i) hepatosplenomegaly with fatty change in the liver, ii) bulky pancreas particularly at the head region with surrounding peri pancreatic fat stranding and collection with thickening at right anterior conal fascia and lateral conal fascia—suggested acute pancreatitis (Fig. 1).

His urine routine examination incidentally reveals protein (++) and ketone (++) without any RBC or RBC cast with FBS 105 mg/dl. His arterial blood gas analysis shows pH 7.25, HCO_3_ 14.9 mmol/l, pCO_2_ 35.3 mmHg, pO_2_ 77.7 mmHg, Na^+^ 140 mmol/L, K^+^ 3.6 mmol/l, Cl^−^ 107 mmol/l, corrected anion gap 18 which revealed that the patient was having high anion gap metabolic acidosis. His serum lactate was 1.13 mmol/l.

Initially, he was managed conservatively with normal saline and antipyretics as per dengue management protocol. After being diagnosed with acute pancreatitis, we introduced Ryle’s tube for continuous drainage and were treated with ringer lactate, antipyretics, proton pump inhibitors, antispasmodics, antiemetics, and opioids. During the hospital stay, this patient was having improved platelet count and became clinically stable. There was no need for a platelet transfusion for the patient. Amylase and lipase levels are gradually decreasing along with other deviant hematological parameters. Eight days after being admitted, he was released in stable condition, and currently, he is fine on follow-up.

## Discussion

DEN-1, DEN-2, DEN-3, and DEN-4 are four antigenically distinctive serotypes belonging to the *Flaviviridae* family and are the agents of DF [5]. DHF and classic DF are clinical illnesses that may or may not be associated with shock. It consists of 3 phases febrile, critical, and recovery phase. The critical phase can be manifested by tachycardia, hypotension, acidosis, organ failure, thrombocytopenia, and disseminated intravascular coagulation (DIC) [6]. Our patient had tachycardia, deranged LFT, thrombocytopenia, euglycemic pancreatic ketoacidosis, and acute pancreatitis. After eight days, he eventually recovered from that phase.

Dengue fever can involve all age groups without any gender predilection. Children have milder symptoms than adults. Clinical features of classic DF are high-grade fever with chills, retro-orbital pain, backache, headache, generalized malaise, weakness, and abdominal pain. Thrombocytopenia, rash, purpura, and organ failure can be seen in critical phases of DHF [7]. Numerous atypical DF presentations have been reported in the literature, including those that are neurological (encephalopathy, acute motor weakness, seizures, Guillain-Barre syndrome, acute viral myositis, acute encephalitis), hepatic (acute hepatic failure, coagulation disturbances), cardiac (myocarditis, sinoatrial block, atrioventricular dissociation), systemic lupus erythematosus, uveitis, acute kidney injury, acute inflammatory colitis, hemophagocytic syndrome, pancreatitis etc [8].

A rare atypical manifestation of dengue is pancreatitis. The diagnosis of AP is most often established by the presence of two of the three following criteria: (i) abdominal pain consistent with the disease, (ii) serum amylase and/or lipase greater than three times the upper limit of normal, and/or (iii) characteristic findings from abdominal imaging (strong recommendation, moderate quality of evidence) [9]. Based on these diagnostic criteria, our patient was diagnosed with acute pancreatitis. The precise pathophysiology is yet unknown. However, it is suggested by one of the postulates that direct virus invasion can lead to swelling and lysis of acinar cells of the pancreas; another postulate suggests that cytokine released by DENV infection can determine the severity of the disease. Mast cells, macrophages, and lymphocytes trigger an inflammatory response through chemokines and cytokines that result in thrombocytopenia, plasma leakage, and increased vascular permeability. Interleukin 2 (IL-2), macrophage migration inhibitory factor (MIF), tumor necrosis factor α (TNF-α), monocyte chemotactic protein-1 (MCP-1), high mobility group box-1 (HMGB-1) and interleukin-8 (IL-8), are among the cytokines that were found to be elevated in DHF and have been linked to causing plasma leakage [10–12].

Our patient also developed ketonuria and ketoacidosis without having any history of diabetes but we couldn’t measure serum ketone values due to an unavailable facility. Mostly ketoacidosis occurs due to uncontrolled diabetes mellitus, prolonged starvation, or acute alcoholism. Our patient was non-alcoholic and non-diabetic without having any prolonged starvation before the onset of this disease. High anion gap acidosis with elevated serum ketone bodies, which include acetone, beta hydroxyl-butyrate, and aceto-acetate in the presence of normal blood glucose level is the feature of euglycemic pancreatic acidosis. It is related to increased peripheral adipolysis with increased serum lipase due to acute pancreatitis. However, acute pancreatitis without diabetes mellitus that results in ketoacidosis is an extremely uncommon manifestation brought on by elevated pancreatic lipase levels in the bloodstream [13, 14].

In summary, while pancreatitis is an uncommon symptom of dengue fever, it is useful to measure and track serum lipase and amylase levels as well as undergo abdominal imaging to rule out acute pancreatitis in patients experiencing abdominal pain. ABG analysis and urine ketone bodies can be done to rule out further euglycemic pancreatic ketoacidosis. Early detection of this dengue manifestation will help to reduce morbidity and mortality through the implementation of improved management.

## Consent

Written informed consent was obtained from the patient for publication of this case report and accompanying images.
